# Probabilistic sea level rise flood projections using a localized ocean reference surface

**DOI:** 10.1038/s41598-023-29297-2

**Published:** 2023-02-08

**Authors:** Noah Paoa, Charles H. Fletcher, Tiffany R. Anderson, Makena Coffman, Shellie Habel

**Affiliations:** 1 Department of Earth Sciences, School of Ocean and Earth Science and Technology, University of Hawaiʻi at Mānoa, 1680 East‑West Rd., POST Room 821, Honolulu, HI 96822 USA; 2 Institute for Sustainability and Resilience, Urban and Regional Planning and UHERO, University of Hawaiʻi at Mānoa, 2560 Campus Rd., George Hall 112, Honolulu, HI 96822 USA

**Keywords:** Climate change, Environmental sciences, Natural hazards

## Abstract

Projecting sea level rise (SLR) impacts requires defining ocean surface variability as a source of uncertainty. We analyze ocean surface height data from a Regional Ocean Modeling System reanalysis to produce an ocean reference surface (ORS) as a proxy for the local mean higher high water. This method allows incorporation of ocean surface level uncertainty into bathtub modeling and generation of probability-based projections of SLR-induced flooding. For demonstration, we model the NOAA Intermediate, Intermediate-high and High regional SLR scenarios at three locations on the island of Oʻahu, Hawai’i. We compare 80% probability-based flood projections generated using our approach to those generated using the Tidal Constituents and Residual Interpolation (TCARI) method. TCARI is the predecessor of VDatum, the standard method used by NOAA available only for the continental U.S., Puerto Rico, and U.S. Virgin Islands. For validation, ORS pixel values representing the Honolulu tide gauge location are compared to tide gauge observations. The more realistic distribution of daily higher high water provided by ORS improves projections of SLR-induced flooding for locations where VDatum is not available. We highlight the importance of uncertainty and user-defined probability in identifying locations of flooding and pathways for additional sources of flooding.

## Introduction

Acceleration of global mean sea level rise is likely to increase with continued global warming^1,2^. Global mean sea level is projected to rise 0.44–0.76 m to 0.63–1.01 m relative to 1995–2014 by the end of the century under the low and very-high greenhouse gas emission pathways (SSP2-4.5 and SSP5-8.5, respectively) presented by the Intergovernmental Panel on Climate Change^3^. However, a magnitude approaching 2 m by 2100 and 5 m by 2150 cannot be ruled out, as there remains deep uncertainty regarding ice sheet processes^3–9^. Given key uncertainties in ice sheet mass loss^10,11^ and long-term responses to warming^12^, this issue continues to complicate efforts by coastal communities to engage in planning for unique and demanding flooding scenarios^13,14^.

The local expression of sea level rise (SLR) can differ significantly from global mean sea level rise^15–17^. In addition to vertical land motion and spatially varying patterns of ocean heat storage, gravitational effects related to mass loss^18^ produce unique local and regional sea level deviations^19–21^. Additionally, present day 100-year extreme sea level events are projected to occur in many locations around the globe at least once a year by the end of the century, even under only 1.5 °C of warming^22^. To address the issue of physical models not accurately representing all major processes contributing to SLR, Sweet et al.^23^ developed both global mean and local relative scenarios out to the year 2100 providing scenario-specific guidelines for planning and decision-making applications. However, their Low and Intermediate-low scenarios are already exceeded by the observed acceleration of global mean sea level rise^1^. Thus Sweet et al.^23^ Intermediate, Intermediate-high, and High relative sea level rise (RSLR) scenarios are being considered in municipal and infrastructure planning^24^.

The most intuitive consequence of SLR is the flooding of coastal areas as they become submerged below the surrounding ocean^25^. Areas surficially connected to the ocean experience flooding as ocean water travels inland across the land surface of low-lying topography or through existing waterways such as drainage systems. This type of flooding has been previously referred to as direct marine inundation^26^ and is commonly simulated using “bathtub” modeling, which uses a digital elevation model (DEM) to characterize locations situated below a projected sea level. This type of modeling ignores dynamic oceanographic processes and thus has also been referred to as “passive”^27^, “static”^28^, “hydrostatic”^29^, “planar”^30^, and “equilibrium”^31^. Relative to other flood-modeling methodologies like wave run-up models, bathtub modeling has a low computational cost making it a simple yet powerful tool for producing flood-visualization products. Bathtub modeling is widely used in adaptation planning as coastal planning reference tools and for coastal-land management decisions (e.g., National Oceanic and Atmospheric Administration (NOAA) SLR Viewer; https://coast.noaa.gov/slr/ and Pacific Islands Ocean Observing System (PacIOOS), State of Hawaiʻi SLR Viewer; https://www.pacioos.hawaii.edu/shoreline/slr-hawaii/).

These passive projections of SLR-induced flooding require an assessment of elevation uncertainty related to the terrain as well as the surrounding tidal surface, which are commonly combined to obtain a cumulative vertical uncertainty^32^. Operating under the assumption that the errors associated with the DEM have zero bias, studies have previously used a Gaussian distribution to represent the vertical uncertainty of the terrain^33^. To estimate tidal surface uncertainty for coastlines of the continental U.S., Puerto Rico and U.S. Virgin Islands, NOAA uses VDatum^34^ (a numerical product representing the difference between modeled and observed water levels). However, VDatum has not been made available for the remainder of the U.S. affiliated islands. In the rest of the insular U.S., NOAA uses the Tidal Constituent and Residual Interpolation (TCARI) method^35^, the predecessor of VDatum. The TCARI method projects tidal vertical uncertainty due to astronomical tides, residual water levels and datum offsets with the use observed water levels and historical data from tidal gauges. TCARI spatially interpolates across the available tide gauges in the region using weighted functions that are generated by solving Laplace’s Equation. Projecting SLR-induced flooding with TCARI is accomplished by assigning a standard score value to each pixel of the DEM and determining flooding using a single-tail percentile rank.

**Figure 1:**
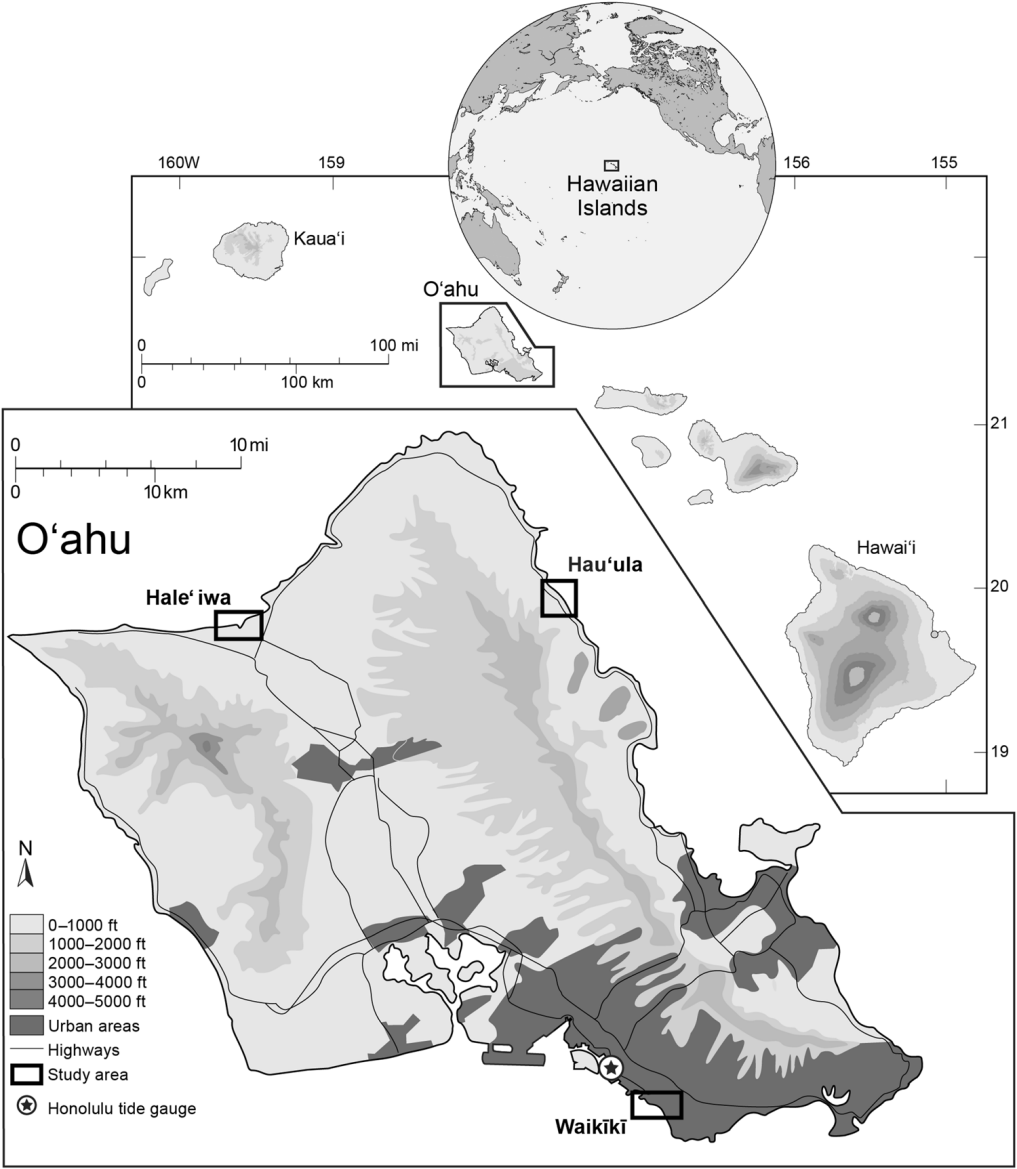
Location of study areas on the island of Oʻahu, Hawaiʻi. Modified from: Fletcher, Mullane and Richmond^40^. Reproduced with permission from the Coastal Education and Research Foundation, Inc.

Here, we present a new method of incorporating the tidal surface uncertainty into bathtub-derived SLR flood projections to provide an improved tool for areas where VDatum is not available. We use reanalysis data of oceanographic conditions produced by PacIOOS with the use of the Regional Ocean Modeling System (ROMS; www.myroms.org)^36^. The PacIOOS reanalysis assimilates observations for the region surrounding the main Hawaiian Islands from a range of sources over the 10-year period 2007–2017 including satellite-derived sea surface temperature, salinity, and height anomalies, depth profiles of temperature and salinity from Argo floats, autonomous Seagliders, shipboard conductivity–temperature–depth and surface velocity measurements from high-frequency radar^37^. We use the ocean-surface height component of the reanalysis, here named an ocean reference surface (ORS), to represent virtual tide stations (individual pixels near the coast) with which we define a tidal surface level and represent spatial variability for the waters around Oʻahu. We produce probability density functions (PDFs) for the modeled daily high-water levels and the DEM uncertainty to develop probabilistic estimates of flood-depth under scenarios of regional SLR. Projecting SLR-induced flooding is accomplished by convolving the PDF of daily higher high-water level of the pixel closest to the area being modeled with a Gaussian distribution of DEM uncertainty. We validate this methodology using data collected at the NOAA Honolulu tide gauge and compare our results with those produced using the TCARI method at three locations on Oʻahu (Fig. 1). Our products are depicted in the form of geographic information systems (GIS) map layers that can be posted to public websites for use by various stakeholders towards developing adaptation plans (e.g., PacIOOS SLR Viewer; https://www.pacioos.hawaii.edu/shoreline/slr-hawaii/). These products consider user-specified flood probabilities, flood depths and specification of flood style for improved use in planning.

## Study sites

The study was based on Oʻahu, the capital island of the Hawaiian Island chain (Fig. 1). Three sites along Oʻahu’s coastal areas were selected with the intent of investigating the performance of the ORS methodology compared to that of the TCARI methodology as well as to compare results across the study sites and potential differences related to their location relative to the Honolulu tide gauge.

Waikīkī: A low-lying coastal area located on the southeastern side of the island, within Honolulu’s primary urban center, bounded by a dredged canal to the north and a heavily engineered, chronically eroding coastline to the south^38^. It sits atop a low-lying coastal terrace which is bounded by the Koʻolau shield volcano to the north, the Leʻahi volcanic tuff crater (commonly known as Diamond Head) to the east and Honolulu’s main seaport and harbor to the west. Waikīkī is the focal point of Hawaiʻi’s tourism industry, hosting the majority of the ~ 6 million tourists that visit Oʻahu annually^39^. Oʻahu’s main tide gauge, the NOAA Honolulu tide gauge, is located ~ 2 mi NW of Waikīkī’s northwestern edge.

Hauʻula: A town which mainly consists of residential properties, is located on the northeastern side of the island, along a NW–SE oriented coastline and at the base of the Koʻolau shield volcano. The low-lying coastal plain where the town is situated is narrower than that of Waikīkī. An intermittent coastal berm bounds the town on the seaward side and the Koʻolau shield volcano to the landward side. The coastline is fronted by a gently sloping fringing coral reef. Kamehameha highway runs along the coastline landward from the berm and is one of only two routes to the north shore of the island from Honolulu’s primary urban center. Hauʻula is located ~ 22 mi N of the Honolulu tide gauge.

Haleʻiwa: The main town of Oʻahu’s north shore, is located between the Waiʻanae mountains and the Koʻolau shield volcano along a NE-SW oriented coastline. This area is comprised of residential properties, farmlands, small shopping centers and other tourism-related infrastructure. Poamoho stream runs from the Waiʻanae mountains, through the town and into the ocean. Similarly, the Helemano stream runs from the Koʻolau shield volcano, through town and meets the Poamoho stream at the mouth. This area also features many smaller water flows associated with agricultural activity. Haleʻiwa is located ~ 25 mi NW of the Honolulu tide gauge.

## Results

### Validation of daily higher high-water distribution

**Figure 2:**
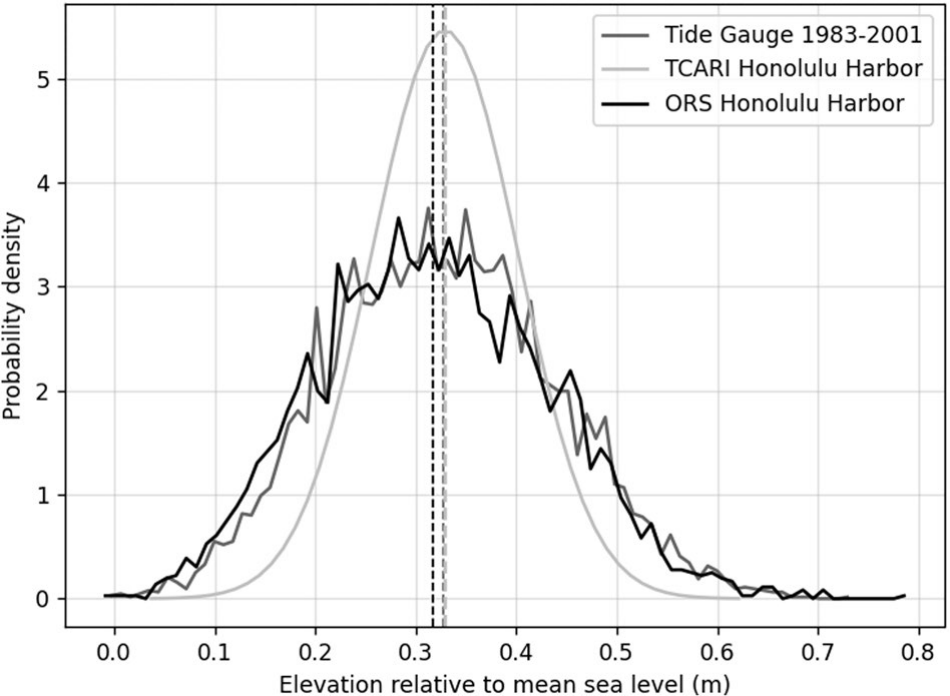
PDFs of daily higher high-water variability as obtained from the Honolulu tide gauge, the ORS, and a normal distribution of the mean of daily higher high water from the TCARI tidal surface. Vertical lines correspond to the mean of the respective distributions.

PacIOOS serves the ROMS reanalysis dataset as a regional pixel network with 4 km resolution. For validation, we treated the pixel closest to the Honolulu tidal gauge as a virtual tide gauge and compared the mean sea level calculated from the ORS to the mean sea level of the Honolulu tide gauge for the tidal epoch (zero) and found that the ORS is 0.013 m higher. The standard deviations from both data sets are very comparable; 0.195 m and 0.191 m for ORS and the Honolulu tide gauge, respectively. The root mean square error between the sea level distributions from the ORS and the Honolulu tide gauge is 0.07 m. The mean higher high water (MHHW) value calculated from the ORS is 0.312 ± 0.115 m, while the Honolulu tide gauge MHHW value is of 0.327 ± 0.112 m above mean sea level. A visual comparison of the distributions of daily higher high water from the Honolulu tide gauge, the ORS and the TCARI grid is provided in Fig. 2.

### TCARI vs ORS

Averaging the flooded-pixel count considering each NOAA SLR scenario using the ORS method reveals that flooding expands from 1.5% of the total mapped area in 2050 to 37.7% by the end of the century. The TCARI method yields similar results with flooded areas expanding from 1.73% to 39.6% between 2050 and 2100. However, there are differences in flood extent and style when comparing the two methods.

**Figure 3:**
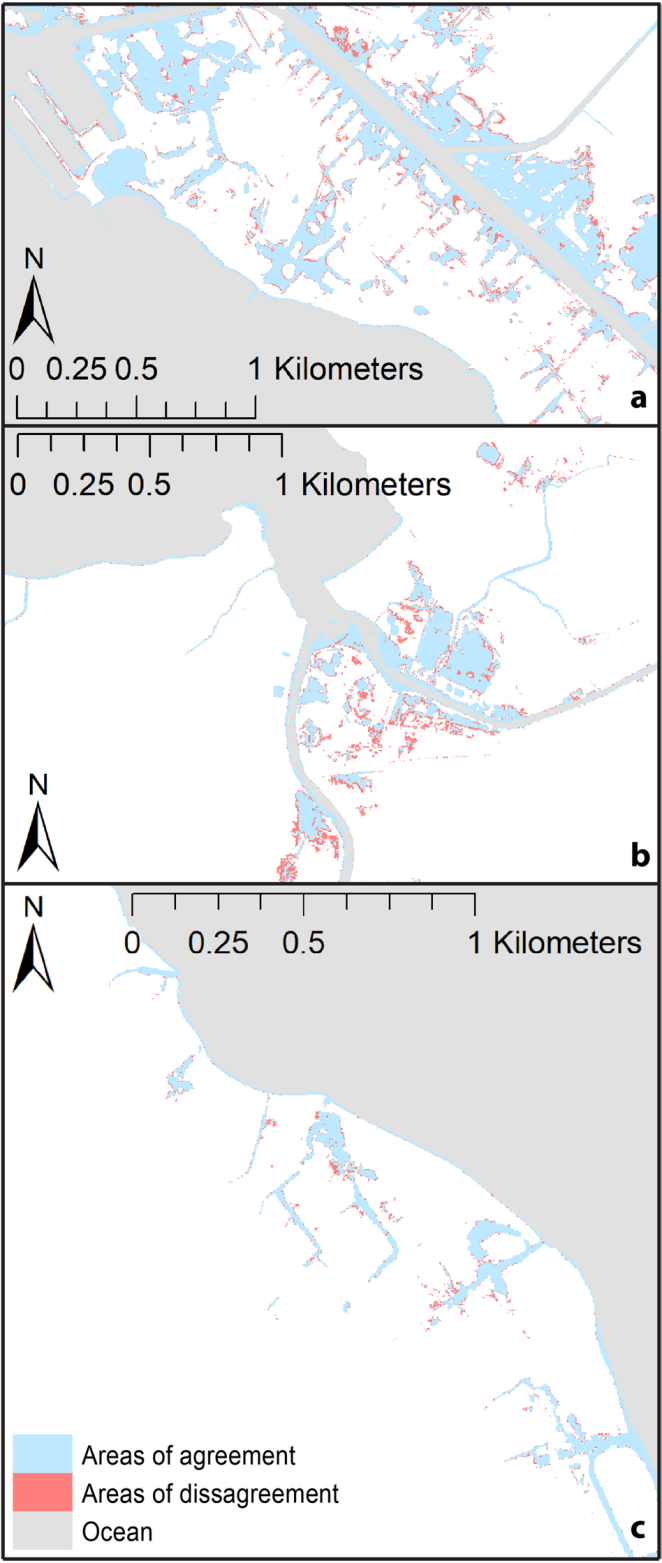
Maps depicting flooding considering the NOAA Intermediate scenario by 2100^23^ for the Honolulu tide gauge (1.19 m) as projected using TCARI and ORS methods; red—area of difference, blue—area of agreement. (**a**) Waikīkī; (**b**) Haleʻiwa; (**c**) Hauʻula. (Maps generated with ArcGIS Desktop 10.8.2; http://www.esri.com).

In general, the TCARI method projects greater total flooding than the ORS method. Areas of difference, due to greater TCARI flooding, tend to surround areas of agreement (as a “fringe” that expands flooding) (Fig. 3). Areas of difference are particularly apparent in Waikīkī and Haleʻiwa but not in Hauʻula.

Table 1 compares TCARI and ORS flood projections. Expressed as a percent difference in flood area, we find substantial disagreement in both total flood area, as well as the type of flooding (surficially connected vs. topographically isolated). The largest disagreement in total flood area is found in the Intermediate scenario where the two methods differ by 26.83% in 2050 and 15.82% in 2100. It is notable that in 2050 there is a 46.84% difference under the Intermediate scenario for flooding at topographically isolated locations. The greatest disagreements are found in the Intermediate scenario by 2050 likely due to the small amount of total flooding projected. Thus, smaller differences between the two methods out of an already small proportion of area flooded result in large discrepancies. Strong agreement is found however, in projecting the flooding of surficially connected areas under the Intermediate-high scenario for 2050 (4.67%) and for 2100 (4.89%). Overall, the strongest agreement in flood projections is found under the High scenario in 2100 where the two methods differ by only 2.64% for surficially connected areas and 2.16% in total flood area. Since the projected area flooded is greater under the High scenario, especially by 2100, we would expect small differences between the two methods to show less discrepancies out of the total flooded area.

**Table 1:** Average across all study sites of percent difference in flood area between the ORS and TCARI approaches, for three NOAA regional sea level scenarios (Honolulu tide gauge), in the years 2050 and 2100.

RSLR scenarios	2050			RSLR scenarios	2100		
	Surficially connected (%)	Topo isolated (%)	Total (%)		Surficially connected (%)	Topo isolated (%)	Total (%)
Intermediate (40 cm)	26.27	46.84	26.83	Intermediate (119 cm)	15.19	16.00	15.82
Intermediate High (57 cm)	4.67	18.29	9.17	Intermediate High (193 cm)	4.89	19.50	5.23
High (75 cm)	12.59	18.58	15.10	High (270 cm)	2.64	19.06	2.16

### Probability-based and flood-depth maps

**Figure 4:**
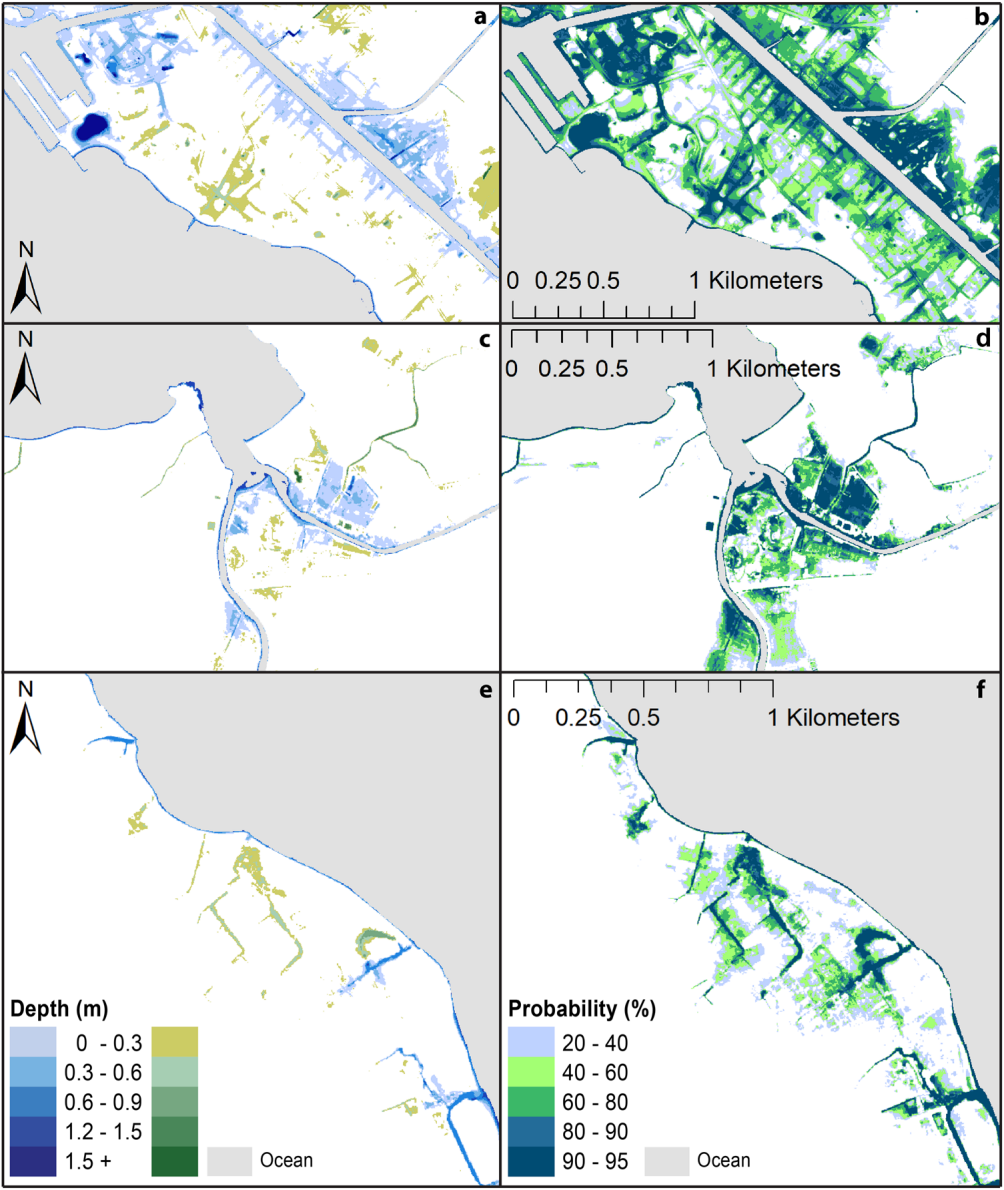
Maps depicting flooding under the Intermediate scenario by 2100 of Sweet et al.^23^ for the Honolulu tide gauge (1.19 m) using the ORS method. (**a**,**c**,**e**) Show the range of flood-depths with at least 80% probability of occurrence for Waikīkī, Haleʻiwa and Hauʻula, respectively (blues—surficially connected; greens—topographically isolated). (**b**,**d**,**f**) Show probability ranges for lands experiencing any amount of flooding in Waikīkī, Haleʻiwa and Hauʻula, respectively (distinction between surficially connected and topographically isolated not identified). (Maps generated with ArcGIS Desktop 10.8.2; http://www.esri.com).

Spatial information regarding flood probability and flood depth are highly useful for various planning applications. User-defined probabilistic flood maps have the potential to streamline adaptation efforts, thus making it important to provide to users as part of interactive websites that host such geospatial information. The cumulative density functions (CDFs) produced following the convolution of the distributions of daily higher high-water level from the ORS and the Gaussian distributions of DEM uncertainty depict the probability of a certain flood depth. Thus, the CDF curves, individually generated for each pixel of the DEMs, can be used by: (1) selecting a probability threshold and finding the corresponding flood-depth value to generate maps depicting flood depth ranges (Fig. 4a,c,e), and (2) selecting a flood-depth value and finding the corresponding probability threshold to generate maps depicting various probability ranges for a single flood depth (Fig. 4b,d,f).

### Flooding style

**Figure 5:**
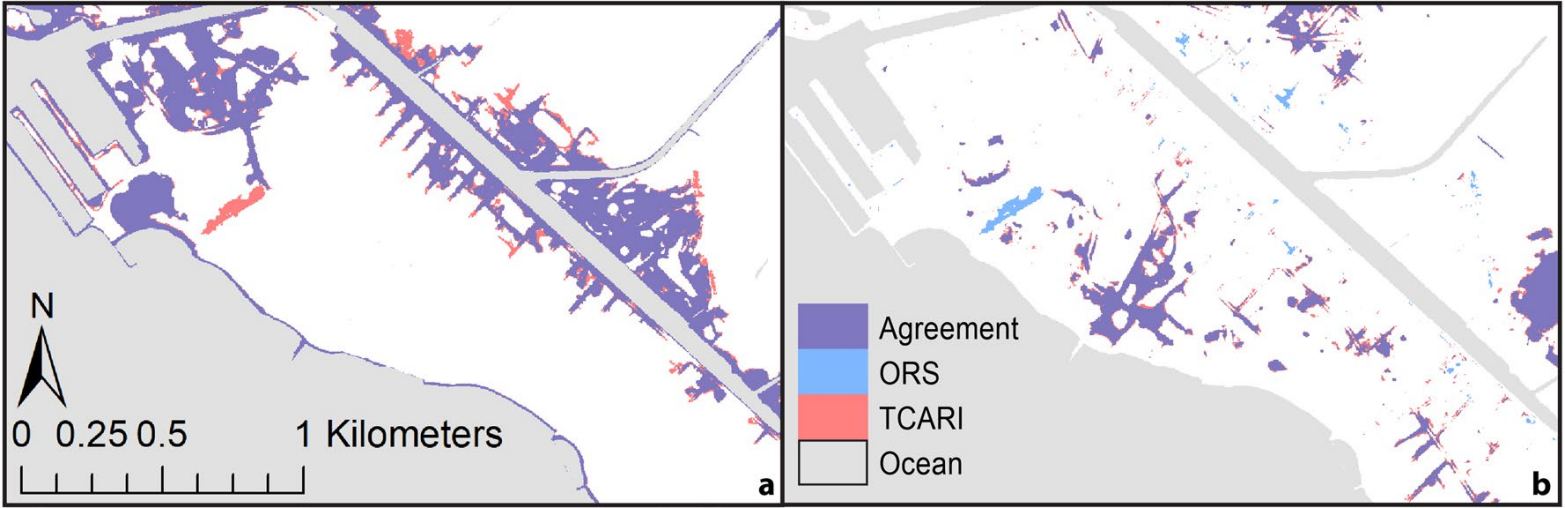
Maps depicting flooding in Waikīkī considering the NOAA Intermediate scenario by 2100^23^ for the Honolulu tide gauge (1.19 m) distinguishing between (**a**) surficially connected and (**b**) topographically isolated; purple—area of agreement, blue—ORS method only, red—TCARI method only. (Maps generated with ArcGIS Desktop 10.8.2; http://www.esri.com).

**Figure 6:**
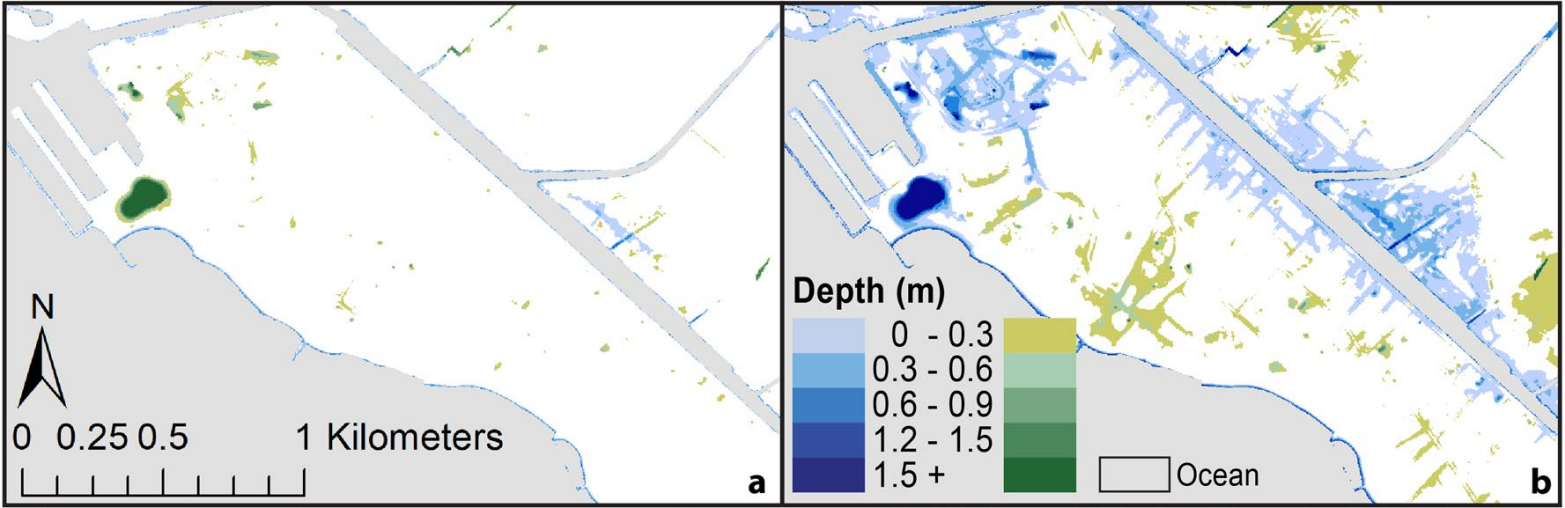
Maps depicting flood depth ranges with at least 80% probability in Waikīkī, (**a**) RSLR flooding scenario by 2050 (High scenario, 0.75 m); (**b**) RSLR flooding scenario by 2100 (Intermediate scenario, 1.19 m) as projected using ORS method (blues—surficially connected; greens—topographically isolated). (Maps generated with ArcGIS Desktop 10.8.2; http://www.esri.com).

When decomposing the total area flooded into topographically isolated and surficially connected flooding, we notice differences in the style of flooding derived with the two methods. Areas that are classified as topographically isolated flooding by one method can be classified as surficially connected by the other method (Fig. 5). Figure 3 depicts the difference between the two methods in terms of total flooding while Fig. 5 depicts the total flooding split into its two components. The projected style of flooding varies depending on the topography of the terrain and the amount of RSLR in a single mapping method. For instance, using the ORS method, in the Waikīkī study area, with a RSLR of 0.75 m by 2050 (High scenario), projected flooding is largely confined to topographically isolated locations, and is not the result of overland marine flow (Fig. 6a). However, by 2100, under the Intermediate scenario (1.19 m), the situation changes and surficially connected, and topographically isolated areas display similar amounts of flooding (Fig. 6b).

## Discussion

Our results show that when comparing ORS model products with flood maps produced using the TCARI method, differences in flooding style and extent are found when considering Sweet et al.’s scenarios for 2050 and 2100. The flood maps reveal several features to consider when applying these products.

### Disagreement

**Figure 7:**
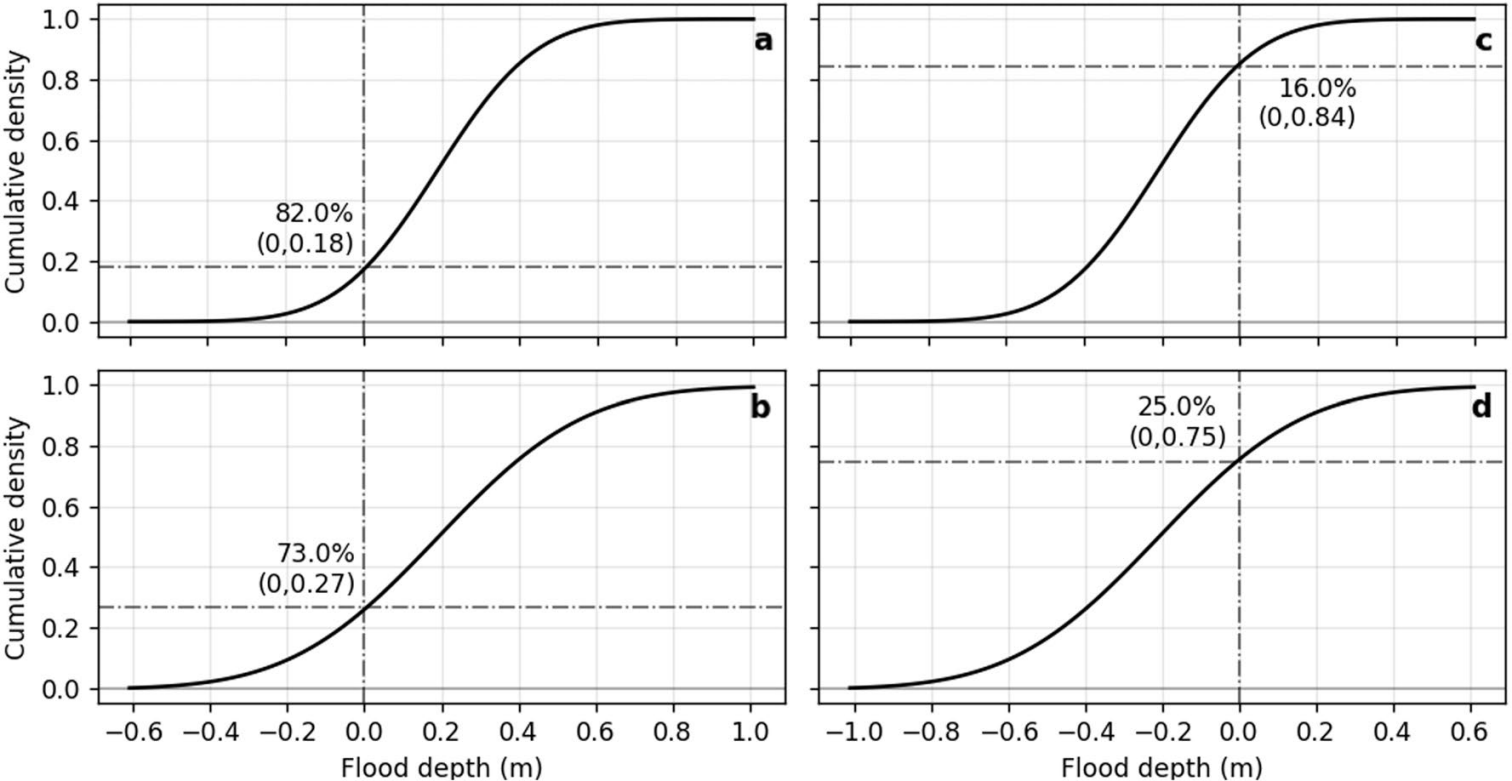
Comparison of two pairs of CDFs with the same mean but different flood-depth spread. CDF pair 8a and 8b illustrate differences that would result in an at least 80% probability-based map. CDF pair 8c and 8d show the result of a probability-based map of at least 20%. The figure illustrates how the shape of the CDF affects probability for a given flood depth.

Analyzing the TCARI and ORS model products highlights a different pattern in the form of a “fringe” surrounding flood projections where the two methods otherwise agree. We find that the fringe zone is a TCARI product. That is, the ORS method projects less flooding than the TCARI method. As shown in Fig. 2, this disagreement reflects a difference in the shape and location of the probability distributions of the ORS and TCARI methods. The TCARI tidal distribution has a tighter spread (higher daily high-water values occur more often), and it is shifted right compared to the ORS distribution. As a result, the CDF obtained using the TCARI method has a steeper slope consequently projecting a larger amount flooding at higher probability values (Fig. 7).

Figure 7 illustrates how the spread of the CDF determines the inundation probability value for a given pixel despite having the same mean. When selecting the value that corresponds to any amount of flooding (i.e., > 0 m flood-depth) the extent of flooding will be lower at higher probabilities when the spread is larger (Note: (b) = 1–0.27 = 0.73 < (a) = 1–0.18 = 0.82). Thus, if we were to map flooding with a probability of at least 80%, CDF (a) would represent a flooded pixel, whereas CDF (b) would not. Conversely, at lower probability values, a steeper CDF (Fig. 7c) would map less flooding than a gentler CDF (Fig. 7d).

### Flood Patterns

The distinction between direct marine flooding and groundwater flooding hinges on whether a flooded area is surficially connected to the ocean. The difference may be determined by a single pixel that allows for a region identified by the ORS method as topographically isolated, to be mapped by the TCARI method as flooded by marine overland flow (Fig. 5). Given the difference in the probability distributions of the TCARI and ORS data, there is considerable opportunity for this to occur. Single pixels or a small group of pixels can connect otherwise isolated areas and may serve as tipping points or flood pathways that open areas to flooding by marine overland flow. The distinction between flooding styles is imperative as low-lying inland areas will likely flood, if not by marine overland flow, by groundwater inundation^26,41^ and addressing each flood style will require additional flood management considerations.

### Distance from tide gauge

When comparing the total area flooded using the ORS and the TCARI methods considering Sweet et al.’s^23^ Intermediate scenario, we observe larger disagreement in Waikīkī and Haleʻiwa (~ 2 mi SE and ~ 25 mi NW from the Honolulu tide gauge respectively) than in Hauʻula (~ 22 mi N from the Honolulu tide gauge) (Fig. 3). These differences suggest that the distance to the Honolulu tide gauge does not play a meaningful role controlling the disagreement between the two methods. ROMS reanalysis includes more thoroughly the observed ocean processes around the main Hawaiian Islands when compared to the TCARI method which interpolates a surface using only tide gauge observations. This results in the ORS daily higher high-water level distribution to resemble more that of the Honolulu tide gauge than the interpolation produced for the TCARI method despite the mean being 0.015 m lower. Hence, the maps that result from the ORS method may be considered a better representation of the amount of flooding projected. In areas of the insular U.S. other than Puerto Rico and U.S. Virgin Island and locations around the world with sparse tidal observations it is appropriate to use ROMS reanalysis data as a source of ocean surface uncertainty.

### King tides

The hydrostatic projections presented in this study do not consider dynamic ocean processes such as wave overtopping, wave run-up and coastal erosion. However, they are useful for the visualization of extreme tidal impacts owing to consideration of tides in the ORS daily higher high-water level distribution. For instance, Thompson et al.^42^ project that by midcentury, coastal sites will see a dramatic increase in king tide frequency. Additionally, under the NOAA Intermediate scenario, global mean sea level is projected to reach 0.3 m by 2050^23^. Thus, a hydrostatic map depicting 0.6 m of flooding is useful for illustrating a 0.3 m king tide on top of the 0.3 m SLR projection.

### Probability-based maps

The range of probability values provided through our method can be assigned as standards to specific types of assets based on their economic value, societal role, or other criteria. For assets of greater value that when flooded impose a larger impact on a community, decision makers should prefer a smaller probability value. Although it might be counter intuitive, a smaller probability value will result in a larger area of projected inundation and therefore a more conservative approach to policy development and decision-making. Put in perspective, areas of at least 80% probability of flooding are surrounded by areas of at least 20% probability of staying dry. Conversely, areas of at least 20% probability of flooding are surrounded by areas of at least 80% probability of staying dry. Some users tend to prefer probability values that correspond to standard deviations in a normal distribution (e.g., 68%, 95%, 99.7%). Similar values were used by Mastrandea et al.^43^ when developing the likelihood scale presented in the fifth assessment report by the Intergovernmental Panel on Climate Change. Other users may prefer different values as thresholds when determining risk, exposure, vulnerability, and other criteria. For instance, NOAA uses 20% and 80% as confidence bounds in their flood mapping methodology^44^. However, information regarding confidence is not immediately visible in NOAA SLR Viewer maps. In addition, the flooded areas are displayed with blue shades but there is no flood-depth value associated with the colors. Although there is uncertainty associated with this type of mapping, the lack of flood-depth information leaves users, particularly those that oversee adaptation of public infrastructure, with poor understanding of potential damage related to flood depth. For instance, a 15 cm flood, which can be associated with a king tide event, is considered a critical threshold for transportation engineering as it is considered likely to stall small vehicles^33,45^. However, a 15 cm temporary flood in open spaces or recreational areas might not be a reason to trigger expensive and disruptive adaptation efforts.

## Conclusion

In this study we have introduced a new methodology for generating flood maps. We use data from a ROMS reanalysis to add the uncertainty of ocean surface height to the uncertainty related to the DEM of the terrain. We use this approach to model three NOAA RSLR scenarios^23^ for the purpose of demonstration and compare the results with the NOAA standard methodology applied to areas of the insular U.S. We find great agreement in the extent of flooding but disagreements in the style of flooding in some areas. The differences arise due to the location and shape of the ORS daily higher high-water level distribution which is more like the distribution of observed sea levels from the Honolulu tide gauge and likely result in more accurate projections. Thus, we find that the ORS method is an improvement over the current method used for the insular U.S. Visualizing the differences between ORS and TCARI simulations reveals the importance of single pixels (or groups of pixels) that create a direct connection to the ocean in otherwise topographically isolated areas. Distinguishing between flood styles is crucial as it can be used to inform adaptation management projects.

Further, we illustrate that bathtub simulations can offer additional valuable information including both flood depth at a given probability threshold, and probability range for a given flood depth. Using the presented method, map servers could be reconfigured to depict flood exposure and allow for a range of user-defined values to assist in optimizing SLR adaptation and management decisions. In doing so, users of said maps would have the ability to define SLR magnitude and probability parameters based on individual needs and identify flooding style and flooding pathways for use in flood management and planning applications.

## Methods

To compare the ORS and TCARI methods we modeled RSLR exposure under the Intermediate, Intermediate-high, and High scenarios of Sweet et al.^23^ for the years 2050 and 2100 for three areas on Oʻahu: (1) Waikīkī (south shore), (2) Hauʻula (east shore), (3) Haleʻiwa (north shore). In Sweet et al.’s^23^ study, RSLR scenarios were produced for all NOAA tide gauge locations in the U.S. The RSLR scenarios consider site-specific influences from phenomena including ocean circulation patterns, changes in Earth’s motion, flexure of the crust and upper mantle due to melting of land-based ice, vertical land motion due to glacial isostatic adjustments, sediment compaction, and groundwater and fossil fuel withdrawals^23^. We recognize that the most recent NOAA report entitled “Global and Regional Sea Level Rise Scenarios for the United States”^46^, provides RSLR projections for Hawaiʻi that slightly differ from those published previously by Sweet et al.^23^ Because SLR is an ongoing phenomenon, these differences in RSLR projection represent an offset in timing towards reaching specific magnitudes of SLR. Since the study’s aim is to characterize flooding extent, depth, and uncertainty associated with specific magnitudes of rise, the use of Sweet et al.’s projections remain appropriate for the purpose of demonstration.

Surficially connected locations exposed to direct marine inundation, and topographically isolated locations vulnerable to groundwater inundation and drainage flooding^47^, are depicted relative to an ocean reference surface that is a proxy for MHHW. We convolved a distribution of the daily highest water level provided by the PacIOOS ROMS reanalysis, with a Gaussian distribution of terrain elevation unique to each DEM pixel to produce a flood-depth probability distribution. The convolution was solved numerically using Python 3.5. The result is a PDF of flood-depth for each pixel of the DEM. This allowed us to produce two types of probability-based flood maps, one that shows the range of depths at a fixed probability of flooding, and one that shows the range of probabilities at a fixed flood-depth. In order to compare the ORS methodology with the TCARI methodology, we produced flood maps illustrating daily higher high-water flooding representing at least 80% probability for a given scenario using both methods. We averaged differences in the flooded-pixel count for each method as well as for the specific cases of surficially connected and topographically isolated locations. Each step is detailed below.

### Ocean reference surface (ORS)

To define an ORS as a proxy for MHHW, we analyzed a 10-year ocean reanalysis for the region surrounding the main Hawaiian Islands^37^ available from the Pacific Islands Ocean Observing System (PacIOOS; https://pacioos.org/). The reanalysis was performed by Powell et al.^37^ using ROMS v3.6 with a 4-D variational data assimilation that includes sea surface temperature, salinity, height anomalies, surface velocity, and depth profiles of temperature and salinity. The output consists of 3-hourly data, at a spatial resolution of approximately 4 km.

To match the datum of the DEM, we followed the procedure used by the U.S. Army Corps of Engineers by subtracting the geoid offset (0.633 m) from each of the ROMS reanalysis pixels^48^. We compared the hourly and 3-hourly means of the Honolulu tide gauge for the time period 2007–2017 and found that the 3-hourly mean is 0.022 m lower than the hourly mean. We added this value to the ROMS data to account for the inherent subsampling bias of the ROMS reanalysis. To examine the validity of the use of ROMS data, we analyzed the daily higher high-water level distribution from the ocean surface height component of the ROMS reanalysis for the location closest to the Honolulu tide gauge (NOAA #1612340) and compared it to the Honolulu tide gauge observations^49^.

The purpose of using ROMS reanalysis data is to generate a distribution of daily higher high-water levels to convolve with a normal distribution of DEM error, not to use the water levels of the ROMS period (2007–2017) as a reference for future sea level rise increments. Therefore, we compared the Honolulu tide gauge MHHW for the NOAA 19-year tidal epoch (1983–2001) to the MHHW from the period (2007–2017). We found a difference of 0.027 m, which was removed from each ROMS data point to account for the difference in mean sea level between time periods. Since our interest is identifying flooding considering mean higher high tide conditions, we scanned the adjusted ocean surface component of the ROMS reanalysis data for daily higher high-water level using a 24-hr window. We calculated the root mean square error between the ROMS adjusted daily higher high-water level and the daily higher high-water level observations from the Honolulu tide gauge for the NOAA tidal epoch. The sea level data contained in each pixel surrounding Oʻahu was similarly adjusted. For modeling, we selected the pixel of the ROMS reanalysis located closest to the area of interest.

### Digital elevation model (DEM)

A DEM representing the topographic elevation of the local terrain was obtained from NOAA Digital Coast^50^. Elevation data used for DEM production was derived from Light Detection and Ranging data collected for the island of Oʻahu in 2013 by the U.S. Army Corps of Engineers^50^. The DEM is a hydro-flattened, bare-earth product referenced to local MSL with a horizontal resolution of 3 m and a vegetated vertical accuracy of 0.268 m^50^. The vegetated vertical accuracy is larger than the vertical accuracy value of the bare-earth DEM. In conformity with NOAA SLR Viewer methodology, we used the vegetated vertical accuracy as the uncertainty (standard deviation) in DEM elevations to produce more conservative results. A normal distribution of DEM error was used for convolution and employed under the assumption that errors associated with the DEM have zero bias^33^.

### ORS method

**Figure 8:**
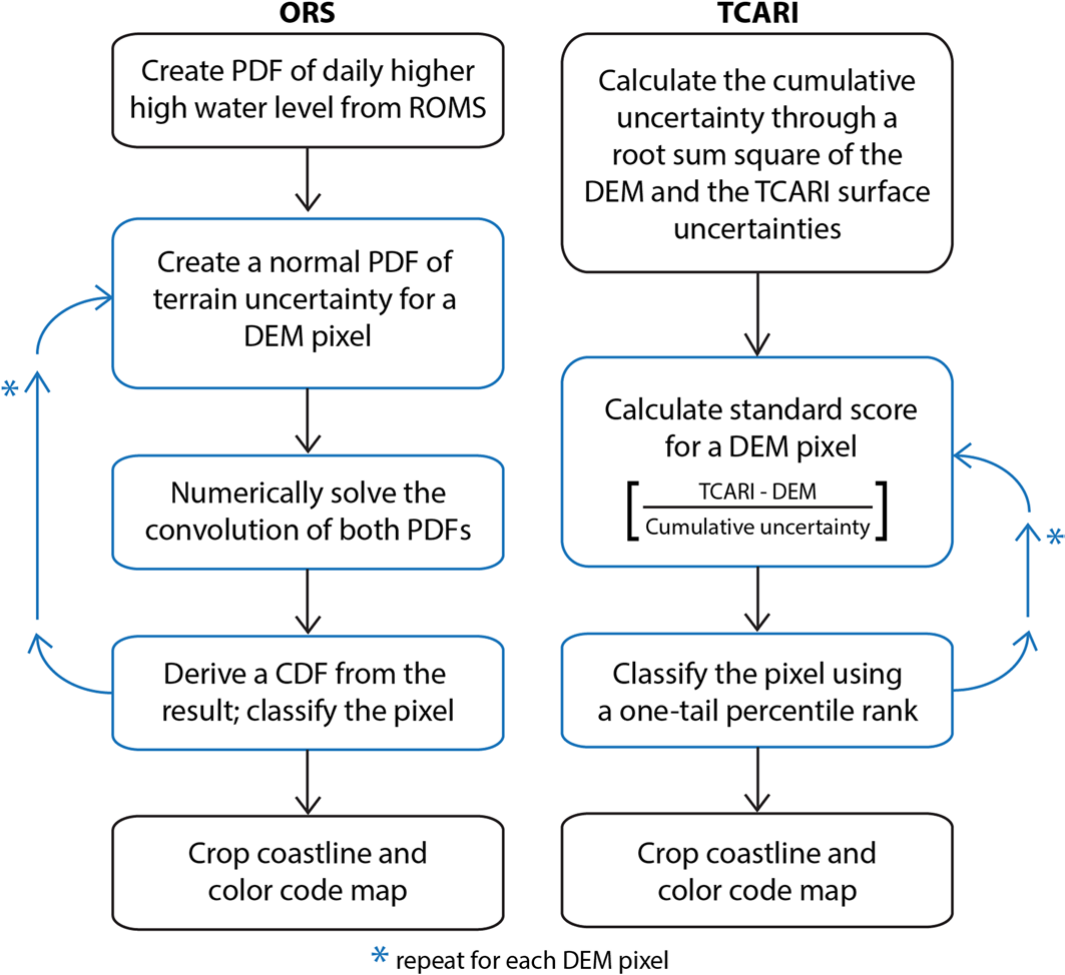
Data-flow diagram comparing the ORS methodology (left) to the TCARI methodology (right); blue boxes are steps to be repeated for all DEM pixels of the modeled area.

Components required to produce bathtub simulations using the ORS method include a distribution of daily higher high-water values and a DEM. The steps followed to perform bathtub modeling using the ORS method are listed below and depicted visually in Fig. 8:

1. Create a PDF of daily higher high-water levels as defined in the ORS.
2. Create a PDF of terrain elevation at each DEM pixel as a gaussian distribution where the mean is the DEM elevation, and the standard deviation is the vegetated vertical accuracy of the DEM.
3. Calculate a PDF of flood depth at each pixel by numerically convolving the PDF of water level (step 1) with the PDF of the DEM (step 2) multiplied by (− 1). Note: the PDF of the sum of two independent random variables is the convolution of the two individual PDFs. Thus, PDF (water level − DEM) = CONV [PDF (water level), PDF (− DEM)].
4. Derive a CDF from the result of the convolution to make two types of maps: a map of flood-depth at a specified probability threshold, and a map of the probability of exceeding a specific flood-depth.
5. To simulate a rise in sea level, add the desired SLR increment to the PDF of the current daily higher high-water levels as defined by the ORS and continue with steps 3 and 4.
6. Once the maps are generated, crop the coastline to visualize the SLR increments in relation to present sea level. The present-day coastline is defined by creating a probability-based flood map following steps provided above without incrementing sea level and selecting the pixels with at least 80% probability of flooding. This raster illustrates the 80% probability of the extent of MHHW. A shapefile is derived from this raster and used to crop the subsequent maps illustrating SLR increments.

### TCARI method

To perform bathtub modeling using the TCARI method, we used the TCARI surface provided by NOAA and the same DEM used in the ORS method. The TCARI surface and the DEM used here are the same as those used in the production of NOAA SLR Viewer maps representing the Hawaiian Islands. The modeling was accomplished by analytically performing convolution of a gaussian distribution representing the pixel value and uncertainty of the TCARI surface and a gaussian distribution representing the pixel value and uncertainty of the DEM. The analytical computation requires the following steps (visually depicted in Fig. 8):

1. Create a single-value cumulative uncertainty surface by calculating the root sum square of the standard deviation of the TCARI surface and the DEM (i.e., sqrt (T^2^ + D^2^); where T^2^ is the uncertainty associated with the TCARI surface and D^2^ is the uncertainty associated with the DEM). The uncertainty associated with the TCARI method is 0.073 m^51^.
2. Calculate a standard score value for each PDF by subtracting the TCARI surface from the DEM elevations for each pixel and dividing by the cumulative uncertainty (step 1) (i.e., z = (TCARI surface − DEM)/uncertainty surface).
3. Classify the pixels using a one-tail percentile rank.
4. As in the ORS method, to simulate a rise in sea level add the desired SLR increment to TCARI surface and continue with steps 2 and 3.
5. As done with simulations produced using the ORS method, crop the simulation produced using the TCARI method using a shapefile representing the present-day coastline.

### Surficially connected vs topographically isolated flood simulation

Modeling and monitoring by Habel et al.^47^ found that groundwater level at high tide in Honolulu’s coastal areas can be represented by the passive MHHW surface. We adopted assumptions made by Anderson et al.^27^ and findings by Habel et al.^47^ that bathtub modeling can be used as a first-cut approach towards identifying vulnerabilities to groundwater inundation owing to similar and opposing magnitudes of the hydraulic gradient and tidal efficiency in coastal groundwater. In depicting flooding related to RSLR, we defined flooded areas as those that probabilistically fall below our reference surface. We then labeled pixels as either: (1) surficially connected to the ocean, or (2) topographically isolated (no surficial connection to the ocean) under the assumption that topographically isolated flooded pixels will be flooded by groundwater inundation. To distinguish between surficially connected and topographically isolated pixels we looked at the intersection between flooded areas for each RSLR scenario and the coastline generated in steps (6) of the ORS method.

To analyze differences in flooded area between TCARI and ORS methodologies, we calculated the percent difference of flooded pixels while distinguishing between flooding styles (surficially connected vs topographically isolated). Note: percent difference = (TCARI area − ORS area)/((TCARI area + ORS area)/2). This was done for each study site and for all NOAA RSLR scenarios. Finally, we averaged percentages for each flooding style across the study sites for 2050 and 2100.

### Uncertainties

Uncertainties considered in both mapping techniques include those associated with the DEM and sea surface level. In the TCARI method, the uncertainties were joined when creating a single value uncertainty surface with a root sum of squares. In the ORS method, the uncertainties were joined when numerically solving the convolution of the daily higher high-water level distribution with the normal distribution of DEM error. When generating flood simulations using either method, we assumed that the magnitude of SLR is known. Therefore, uncertainties related to SLR projections were not accounted for within each modeling method. It should also be noted that our approach of simulating increases in sea level neglects potential changes in tide behavior and storm surge activity.

### Probability of flood-depth

Maps depicting ranges of flood-depth under an assumed RSLR scenario (e.g., 1.19 m by 2100, Intermediate scenario^23^) were produced using chosen probability values (e.g., 20%, 80%, 90%) and by matching depths from the CDF for each pixel. Maps have been color coded to highlight 0.3 m depth increments for the given RSLR scenario, and to identify surficially connected vs. topographically isolated pixels. These provide depictions of flood depths for a given probability at a given SLR scenario, which are useful for land use planning and infrastructure design^52–54^ or further processed to develop risk-management products^55,56^.

### Probability of flooding

Maps that depict the probability of flooding under a given SLR flood depth were similarly derived using the CDF of individual pixels. Here, color coding reveals probability rather than depth. The entire map depicts the probability of surficially connected and topographically isolated flooding produced by a specific magnitude of SLR. Such maps are useful for planning applications, especially where the probability of a specific flood-depth is a critical design parameter, such as in transportation planning^52,54,57^.

## Data Availability

The data used in this study is available from the groups in the text or in citations. Other intermediate products are available upon request to npaoakan@hawaii.edu.
